# PEER-BASED CHECK-IN SUPPORT AND PERSONAL AGENCY IN TANGIBLE INTERFACE DESIGN FOR REDUCING ISOLATION IN OLDER ADULTS

**DOI:** 10.1093/geroni/igad104.1381

**Published:** 2023-12-21

**Authors:** Pallabi Bhowmick, Lesa Huber

**Affiliations:** Indiana University Bloomington, Bloomington, Indiana, United States; Indiana University Bloomington, Bloomington, Indiana, United States

## Abstract

About one in four older adults in the US are socially isolated, an issue worsened by the COVID-19 pandemic. The aims of a 2-week diary study and field deployment of a peer-based tangible check-in system with 16 participants aged 65 to 87 were to understand how older adults check-in with others, who they check-in with, what difficulties they face, and how technology can help them stay connected. Findings show that perceived check-in behavior varies from actual check-in behavior. Although participants listed family as their strongest relationships forming the closest social circle, 75% of the participants had up to third-quarters of their communication with friends as compared to all other relationships. Secondly, passive check-in and monitoring from family members make older adults non-participatory recipients of care and takes away human agency. The check-in system allowed participants to actively check-in on their peers. Participants found the technology non-stigmatizing and bonded with each other quickly. Finally, older adults prefer tangible interfaces, such as buttons and physical controls over digital interfaces, because they do not require fine motor skills or visual acuity. Tangible systems can provide familiar, intuitive, and accessible systems by leveraging everyday physical objects as user interfaces. Study participants found the tangible check-in system easy and enjoyable to use. Such tangible peer-based systems improve social connectedness, address ageism by restoring agency, meet accessibility and aesthetic needs, thereby encouraging technology adoption. They also empower older adults to maintain their independence, instill in them a strong sense of community, and improve quality of life.

